# The application of intravenous general anesthesia under nasopharyngeal airway assisted ventilation undergoing ureteroscopic holmium laser lithotripsy: A prospective, single-center, controlled trial

**DOI:** 10.1515/med-2024-1046

**Published:** 2024-09-23

**Authors:** Xuandong Jia, Min Wang

**Affiliations:** Department of Anesthesiology, The 904th Hospital of the Joint Logistic Support Force of PLA, Wuxi, 214000, Jiangsu, China

**Keywords:** ureteroscopic holmium laser lithotripsy, nasopharyngeal airway, laryngeal mask, intravenous general anesthesia

## Abstract

To observe the effect of intravenous general anesthesia under nasopharyngeal airway-assisted ventilation on patients undergoing ureteroscopic holmium laser lithotripsy. One hundred and twenty patients who underwent ureteroscopic holmium laser lithotripsy in our hospital were selected and randomly divided into nasopharyngeal airway group (research group) and laryngeal mask group (control group). These patients, respectively, received intravenous anesthesia under nasopharyngeal airway and laryngeal mask-assisted ventilation. The following evaluation indexes were compared and analyzed between the two groups, including anesthetic effect, hemodynamics, stress response, postoperative recovery, adverse reactions, etc. There were no significant differences in Visual Analog Scale, hemodynamics, and stress response between the two groups at each time point (*P > 0.05*). There were no significant differences in residence time and postoperative recovery time between the two groups (*P >* 0.05). The difference in airway establishment time between the two groups was statistically significant (*P* < 0.05), and cases with blood in the research group was significantly lower than those in the control group (*P* < 0.05). Patient satisfaction in research group was significantly higher than those in the control group (*P* < 0.05). The clinical effect of intravenous general anesthesia under nasopharyngeal airway-assisted ventilation in ureteroscopic holmium laser lithotripsy is significant, which helps to stabilize patients’ hemodynamics, reduce their stress response and adverse reactions, and improve the satisfaction rate of patient.

## 1 Introduction

The incidence rate of ureteral calculi is increasing year after year with the change of people’s living habits and diet structure [1]. If not treated in time, it may cause ureteral obstruction, hydronephrosis, or pyonephrosis, increasing the probability of ureteral and renal damage, and also increase the difficulty of treatment to a certain extent [2,3]. Ureteroscopic holmium laser lithotripsy has the advantages of minimally invasive, significant effect, and short recovery time, making it a safe and effective way to treat ureteral stones [4]. It has been widely accepted by physicians and patients [5]. However, the anesthesia method for this surgery has always been controversial. If the analgesic and sedative effect is not good during the operation, it can easily lead to patient restlessness, lack of cooperation, and even ureteral perforation, which will seriously affect the prognosis [6]. With the application of accelerated rehabilitation concept in urology, ureteroscopy holmium laser lithotripsy, a minimally invasive surgery, still requires the use of precise anesthesia plan to prevent damage to the function of organ tissues caused by surgical and anesthesia operations during the perioperative period. How to reduce stress response and promote prognosis recovery has gradually become the focus of attention for the above surgery [7]. In the past, spinal anesthesia methods were often chosen, but these methods can easily increase patient discomfort and even lead to complications [4,7]. At present, most surgeries choose tracheal intubation or laryngeal mask general anesthesia in the general anesthesia scheme. Literature [8,9] believed that ureteroscopy holmium laser lithotripsy was relatively more suitable under general anesthesia, and also provided the advantages of laryngeal mask general anesthesia compared to tracheal intubation. However, there are relatively few published studies on the application of intravenous general anesthesia under nasopharyngeal airway-assisted ventilation in ureteroscopic holmium laser lithotripsy. Chen et al. applied the general anesthesia scheme under nasopharyngeal airway-assisted ventilation to retinal laser surgery in low body weight newborns, and compared it with the laryngeal mask group and tracheal intubation group. It was found that the nasopharyngeal airway group had significant advantages in postoperative hemodynamic indicators [10]. Therefore, the application of intravenous general anesthesia under nasopharyngeal airway-assisted ventilation in ureteroscopic holmium laser lithotripsy is a new exploration. This article explores the application effect of intravenous general anesthesia under nasopharyngeal airway-assisted ventilation in ureteroscopic holmium laser lithotripsy by observing indexes such as anesthesia efficacy, hemodynamic parameters, and stress response. The current reports are as follows.

## 2 Information and methodology

### 2.1 General information

One hundred and twenty patients with ureteral stones who underwent ureteroscopic holmium laser lithotripsy at our hospital from January 2023 to December 2023 were selected as the study subjects. Intraoperative anesthesia was performed under intravenous general anesthesia. According to different ventilation modes, patients were randomly divided into a nasopharyngeal airway group (research group) and a laryngeal mask group (control group) using a random number table method, with 60 cases in each group. Inclusion criteria included: (1) complete clinical data, (2) consistent with the diagnosis of upper and middle ureteral stones, with a stone diameter of <1.5 cm, (3) meets the surgical indications for ureteroscopic holmium laser lithotripsy, and (4) there is no history of allergy to the drugs used in the study. Exclusion criteria were (1) individuals with a history of urological surgery or urethral stricture who are unable to undergo endoscopy; (2) severe liver and kidney lesions, concurrent tumors, hemorrhagic diseases, and severe spinal deformities; (3) concomitant mental illness; (4) patients with hypertension, diabetes, and other complications that are not well controlled before surgery; (5) individuals with a history of sleep apnea, nasal malformations, rhinitis, nosebleeds, or nasal surgery, as well as other respiratory diseases; and (6) patients with contraindications to laryngeal mask application such as habitual reflux and other contraindications to anesthesia.

### 2.2 Anesthesia methods

Prior to surgery, all patients were routinely prohibited from drinking or eating. After entering the operating room, the peripheral venous channel was opened to monitor routine indicators such as electrocardiogram, blood oxygen saturation, heart rate, blood pressure, and Bis, and administer oxygen with a mask (5 L/min).

Research group: Patients were given intravenous infusion of 2 mg of hydromorphone hydrochloride injection, and intravenous pump of dexmedetomidine hydrochloride at a loading dose of 1 µg/mL for 10 min. The maintenance dose of dexmedetomidine hydrochloride was changed to 0.2–0.7 µg/(kg h). Intravenous infusion of 10 mg/mL propofol and 10 µg/mL remifentanil, in plasma target control mode, Marsh model, and Minto model were selected, respectively. The target concentrations of propofol and remifentanil were loaded with 2.5–35 and 3.5–4.5 ng/mL, respectively. When the patient’s eyelash reflex disappeared and the anesthesia depth Bis value decreased to 60, the nasopharyngeal airway was inserted, and then the anesthesia machine respiratory circuit was connected through a mask, set to manual control mode, and pure oxygen was inhaled for 5 L/min. The maintenance doses of propofol and remifentanil were changed to 1.5–2.5 and 2.5–3.5 ng/mL, respectively.

The drug concentration has reached its peak at this time and the patient’s breathing condition needs to be observed. If breathing pauses occur, brief artificial ventilation should be given until the patient’s spontaneous breathing is restored. The patient’s spontaneous breathing should be retained during surgery, and the Bis value should be maintained between 50 and 60. During surgery, research group may encounter the following situations: (1) SpO_2_ < 92%, patients should be provided with mandibular support and face mask pressure oxygen supply. If necessary, tracheal intubation is performed. (2) When blood pressure is 20% lower than the preoperative baseline, vasoactive drugs such as ephedrine or hydroxylamine should be administered to patients. (3) Body movement reaction, administer a BOLUS dose of propofol/remifentanil until the body movement reaction disappears.

Control group: Laryngeal mask general anesthesia was used, and patients were intravenously injected with midazolam 0.03 mg/kg, propofol 1 mg/kg, sufentanil 0.5 µg/kg, and rocuronium 0.6 mg/kg for anesthesia induction. After induction, a laryngeal mask (Tampa, single lumen steel wire reinforced type) was inserted. Intravenous infusion of 3–4 mg/(kg h) propofol and 3–4 µg/(kg h) remifentanil was administered for anesthesia maintenance. If there is a leakage or displacement of the laryngeal mask during surgery, resulting in insufficient ventilation and a decrease in SpO_2_, and the patient still does not improve after adjusting the position of the laryngeal mask, deepening anesthesia, or adding muscle relaxants, emergency tracheal intubation should be performed.

### 2.3 Observation indicators

Observation indicators mainly included: (1) Hemodynamics: Using the Mindray BeneView T6 monitor, monitor the hemodynamic parameters of the two groups of patients before anesthesia induction (T0), at the time of insertion of a laryngeal mask or nasopharyngeal airway (T1), and at the time of extraction (T2), including mean arterial pressure (MAP), heart rate (HR), and pulse oxygen saturation (SpO_2_). (2) Stress response: Measure the serum cortisol (Cor) and norepinephrine (NE) levels of patients. Detection method: Before surgery (T3) and 0.5 h (T5), 6 h (T6), and 24 h (T8) after surgery, 3 mL of venous blood was collected from patients, maintained at a rate of 3,000 r/min, and centrifuged for 10 min to obtain serum. The patient’s Cor and NE were detected using Roche electrochemiluminescence method. (3) Anesthesia effect: Evaluate the patient’s awakening time (T4) and subjective pain sensation and retention time at 6 h (T6), 12 h (T7), 24 h (T8), and 48 h (T9) after surgery. Among them, subjective pain perception was evaluated using the Visual Analog Scale (VAS), with a score of 0–10, and the higher the score, the more severe the pain [11,12]. The retention time refers to the time from preoperative preparation to postoperative recovery of the patient in the operating room. (4) Postoperative recovery: The patient’s postoperative recovery time and perioperative satisfaction. Among them, patient satisfaction is measured through a self-made questionnaire survey, which is divided into three levels: very satisfied, satisfied, and dissatisfied. (5) Establishment of airway time: Refer to the time required from placing a nasopharyngeal airway or laryngeal mask to achieving ventilation. (6) Adverse reactions: Including the following situations: SpO_2_ < 92%, body movement, number of unplanned tracheal intubation cases, and number of cases with blood on the surface after removing the airway (used to evaluate mucosal damage, abbreviated as bleeding cases) [13].

### 2.4 Statistical analysis

Statistical data were analyzed using SPSS 22.0 software. The measurement data are represented by mean and standard deviation, and independent sample *t*-test is used for intergroup comparison. In the study, hemodynamic indicators, stress response indicators, VAS scores at different times, retention time, and postoperative recovery time are used as measurement data. The count data are represented by *n* (%), and *χ* ^2^ test is used in intergroup comparison. Patient satisfaction and adverse reactions in the study are the count data. *P* < 0.05 is considered statistically significant.


**Ethical approval:** This study was approved by the Medical Ethics Committee of our hospital.
**Informed consent:** Informed consent form was signed by the patients.

## 3 Results

### 3.1 Demographic data

There was no statistically significant difference in general information such as gender, age, disease course, and stone diameter between the two groups (*P* > 0.05), as shown in Table 1.

**Table 1:** Comparison of general situation between the two groups

Index	Research group (*n* = 60)	Control group (*n* = 60)	*t/χ* ^2^	*P*
Male [*n* (%)]	36 (60.00)	37 (61.67)	0.035	0.852
Female [*n* (%)]	24 (40.00)	23 (38.33)		
Age	41.51 ± 7.56	42.81 ± 8.66	−0.883	0.379
Disease course (month)	21.10 ± 3.16	21.62 ± 2.58	−0.993	0.323
Stone diameter (cm)	1.12 ± 0.46	1.11 ± 0.34	0.252	0.801

### 3.2 Comparison of hemodynamics between two groups of patients

**Figure 1:**
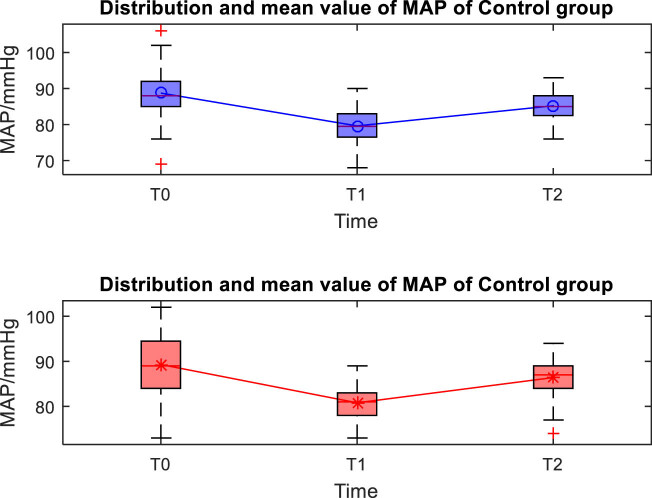
Distribution and mean value of MAP of the two groups.

**Figure 2:**
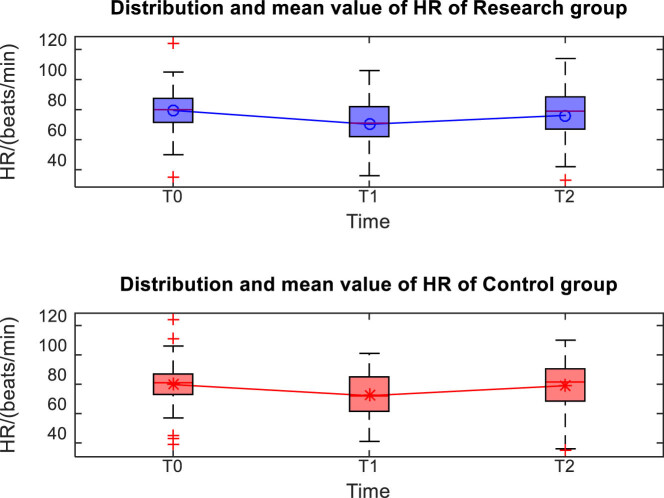
Distribution and mean value of HR of the two groups.

**Figure 3:**
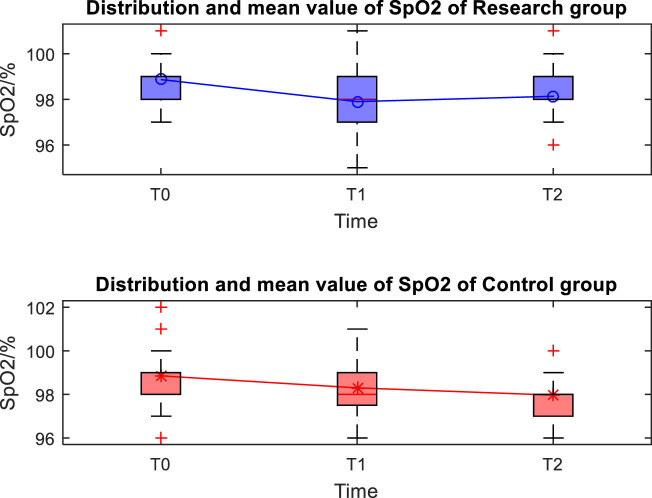
Distribution and mean value of SpO_2_ of the two groups.

The distribution and mean value of HR, MAP, and SpO_2_ of the two groups at T0, T1, and T2 are shown in Figures 1–3. Taking the distribution of the measured values of HR of the S group at T0 as an example, the box diagram contains the upper adjacent, lower adjacent, and median of the distribution of the measured values. The box part represents the interval of most measured values.

The MAP and HR at T1 were significantly lower than those at T0 and T2 (*P* < 0.05), and there was no significant difference in SpO_2_ at each time point (*P* > 0.05) between the two groups of patients. There was no significant difference in MAP, HR, and SpO_2_ between the two groups of patients at different time points (*P* > 0.05), as shown in Table 2.

**Table 2:** Comparison of hemodynamic parameters between the two groups (*x̄* ± *s*)

Index	Research group (*n* = 60)	Control group (*n* = 60)	*t*	*P*
**MAP (mmHg)**				
T0	88.72 ± 6.44^*^	89.31 ± 6.78^*^	−0.482	0.631
T1	79.64 ± 4.41	80.81 ± 3.44	−1.397	0.165
T2	85.21 ± 4.01^*^	86.42 ± 4.21^*^	−1.612	0.110
**HR (times/min)**				
T0	79.51 ± 14.68^*^	79.77 ± 14.84^*^	−0.096	0.924
T1	70.26 ± 15.14	72.19 ± 15.23	−0.679	0.498
T2	76.14 ± 16.03^*^	78.97 ± 15.99*	−0.971	0.334
**SpO**_**2**_ **(%)**				
T0	98.87 ± 0.82	98.81 ± 0.90	0.370	0.712
T1	97.95 ± 1.22	98.34 ± 1.31	−1.209	0.230
T2	98.16 ± 0.90	97.95 ± 0.80	1.312	0.192

Note: Compared to T1 time, *P < 0.05 for other time points within the group.

### 3.3 Comparison of stress responses between two groups of patients

**Figure 4:**
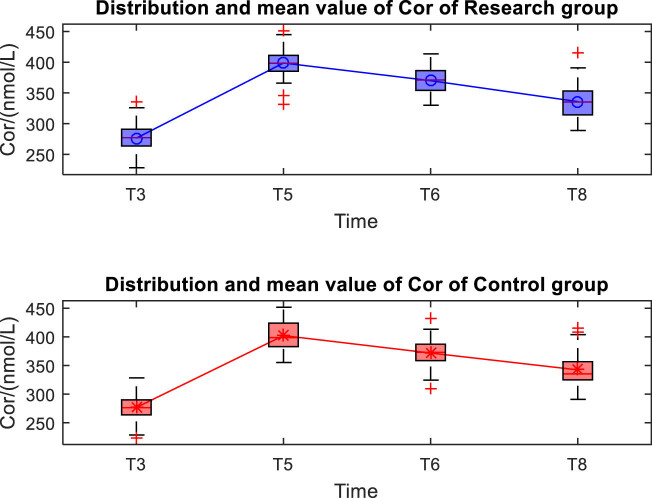
Distribution and mean value of Cor of the two groups.

**Figure 5:**
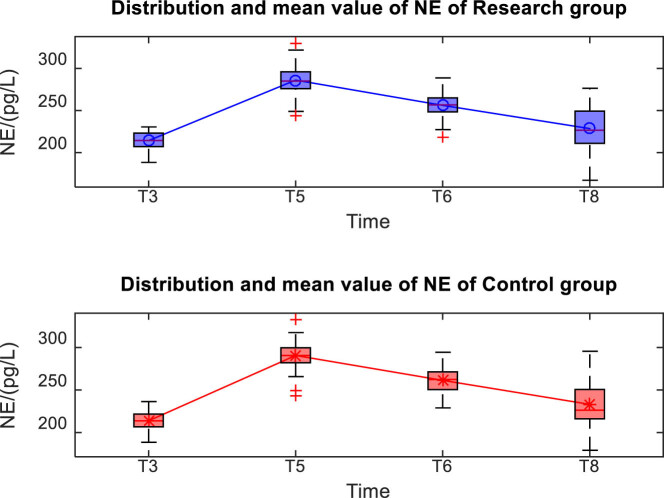
Distribution and mean value of NE of the two groups.

The distribution and mean value of Cor and NE in the two groups after operation are shown in Figures 4 and 5. There was no significant difference in the Cor between the two groups at T3, T5, T6, T8 (*P* > 0.05). Compared with the Cor of patients in both groups in T3, the Cor of patients in both groups significantly increased at T5, T6, T8 (*P* < 0.05). There was no significant difference in the NE between the two groups at T3, T5, T6, T8 (*P* > 0.05). Compared with the NE of patients in both groups in T3, the NE of patients in both groups significantly increased at T5, T6, T8 (*P* < 0.05), as shown in Table 3.

**Table 3:** Comparison of stress responses between the two groups (*x̄* ± *s*)

Index	Research group (*n* = 60)	Control group (*n* = 60)	*t*	*P*
**Cor (nmol/L)**				
T3	276.46 ± 20.59	276.80 ± 20.25	−0.106	0.916
T5	398.67 ± 22.41^*^	402.16 ± 25.24^*^	−0.766	0.445
T6	369.92 ± 21.01^*^	371.73 ± 23.61^*^	−0.470	0.639
T8	335.87 ± 26.50^*^	342.34 ± 27.49^*^	−1.333	0.185
**NE (pg/L)**				
T3	214.32 ± 10.14	213.81 ± 10.89	0.255	0.799
T5	286.12 ± 17.36^*^	290.61 ± 15.83^*^	−1.475	0.143
T6	256.12 ± 13.95^*^	261.35 ± 14.86^*^	−1.949	0.054
T8	228.55 ± 25.44^*^	233.13 ± 27.65^*^	−9.69	0.335

Note: Compared to preoperative time, *P < 0.05 was observed at other times within the group.

### 3.4 Comparison of VAS scores between two groups of patients at different postoperative times

**Figure 6:**
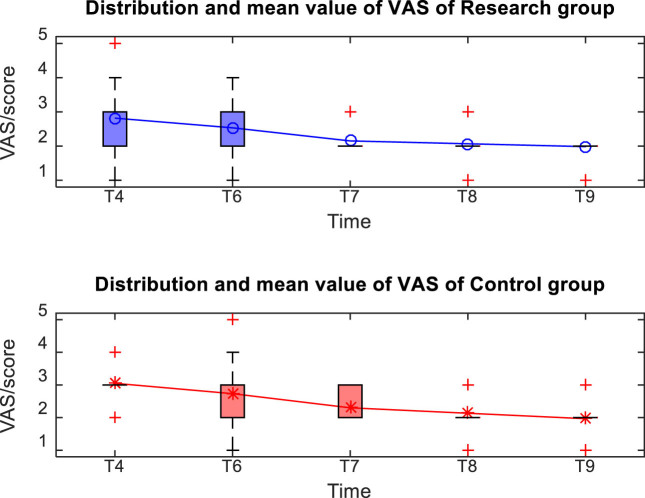
Distribution and mean value of VAS of the two groups.

The distribution and mean value of VAS scores of patients in the two groups after operation are shown in Figure 6. There was no statistically significant difference (*P* > 0.05) in the VAS scores of the two groups of patients at each postoperative time, as shown in Table 4.

**Table 4:** Comparison of VAS scores between the two groups at different time after operation (*x̄* ± *s*)

VAS score	Research group (*n* = 60)	Control group (*n* = 60)	*t*	*P*
T4	2.86 ± 0.59	3.01 ± 0.39	−1.693	0.093
T6	2.56 ± 0.53	2.74 ± 0.75	−1.570	0.119
T7	2.21 ± 0.27	2.32 ± 0.34	−1.021	0.309
T8	2.07 ± 0.47	2.14 ± 0.37	−0.907	0.367
T9	1.89 ± 0.21	1.94 ± 0.47	−0.597	0.552

### 3.5 Comparison of retention time, airway establishment time, postoperative recovery time, and patient satisfaction between two groups of patients

There was no statistically significant difference in the retention time and postoperative recovery time between the two groups of patients (*P* > 0.05). There was a statistically significant difference (*P* < 0.05) in the time and satisfaction of establishing airways between the two groups of patients, as shown in Table 5.

**Table 5:** The retention time, postoperative recovery time, time for airway establishment, and patient satisfaction of the two groups [(*x̄* ± *s*), *n* (%)]

Index	Research group (*n* = 60)	Control group (*n* = 60)	*t/*χ^2^	*P*
Detention time (min)	61.19 ± 15.93	63.71 ± 16.13	−0.861	0.391
Establishing airway time (s)	13.42 ± 2.43	28.37 ± 3.97	−24.774	0.000
Postoperative recovery time (days)	4.77 ± 1.55	4.89 ± 1.54	−0.430	0.668
Patient satisfaction [*n* (%)]	59 (98.33)	53 (88.33)	4.821	0.028

### 3.6 Comparison of adverse reactions between two groups of patients

The number of cases with blood in the control group was significantly higher than that in the research group, and the difference was statistically significant (*P* < 0.05), as shown in Table 6.

**Table 6:** Comparison of adverse reactions between the two groups [*n* (%)]

Index	Research group (*n* = 60)	Control group (*n* = 60)	*χ* ^2^	*P*
SpO_2_ < 92% [*n* (%)]	2 (3.33)	1 (1.67)	0.342	0.559
Physical movement [*n* (%)]	1 (1.67)	0 (0.00)	1.008	0.315
Number of cases with blood [*n* (%)]	3 (5.00)	12 (20.00)	6.171	0.013
Unplanned tracheal intubation *n* [(%)]	0 (0.00)	1 (1.67)	1.008	0.315

## 4 Discussion

Ureteroscopic holmium laser lithotripsy requires high standards for anesthesia. While general anesthesia offers rapid onset, effective anesthesia, and high stone clearance rates, it also brings about delays in recovery, pulmonary complications, and hemodynamic fluctuations. This is especially true for tracheal intubation general anesthesia, which significantly elevates patients’ stress responses. In particular, general anesthesia with tracheal intubation will greatly increase the patient’s stress response [2,3,14]. The surgery and anesthesia method have a significant impact on hemodynamics and stress level of patients. Therefore, optimizing the anesthesia method and selecting anesthetic drugs reasonably can be used to reduce the impact of surgery and anesthesia on hemodynamics and stress level. Research indicates that the use of laryngeal mask general anesthesia in ureteroscopic holmium laser lithotripsy supports stable hemodynamics and reduces stress responses in patients. However, there are also issues such as poor sealing effect, increased oral secretions, and affecting patient comfort [15,16]. Nasopharyngeal airway-assisted ventilation can fully preserve the patient’s autonomous breathing, and its application in intravenous anesthesia can quickly alleviate respiratory obstruction and prevent intraoperative hypoxemia [17]. Therefore, it is worth exploring whether the application of intravenous general anesthesia under nasopharyngeal airway-assisted ventilation in ureteroscopic holmium laser lithotripsy can achieve better results in clinical application.

Related studies have found that the application of propofol remifentanil combined with laryngeal mask ventilation in ureteroscopic holmium laser lithotripsy has a significant anesthetic effect, can maintain patient hemodynamic stability, and help reduce patient stress response [15,16]. The results of this study showed that there were no significant differences in VAS scores, hemodynamic indicators, and stress response indicators between the research group and the control group at various postoperative times. The above results indicate that the application of intravenous general anesthesia under nasopharyngeal airway-assisted ventilation in ureteroscopic holmium laser lithotripsy has a significant effect, which could achieve the same effect as general anesthesia with laryngeal mask and further verifies the conclusions of refs [15,16].

The underlying cause was examined from the standpoint of the anesthesia protocol. The control group employed traditional general anesthesia with laryngeal masks, whereas the research group utilized intravenous general anesthesia supported by nasopharyngeal airway ventilation. Dexmedetomidine and hydromorphone were co-administered during the procedure. As a new type of highly selective α-2 adrenergic receptor agonist, dexmedetomidine can reduce the response of adrenergic nerves to pain stimuli by inhibiting sympathetic nerve excitation, effectively inhibiting patient sympathetic activity, achieving advantages such as pain relief, sedation, and respiratory inhibition. When used in compound anesthesia surgery, dexmedetomidine can effectively improve patient’s awakening quality, buffer blood pressure fluctuations, maintain hemodynamic stability, and alleviate patient stress reactions [18,19]. Hydromorphone is a semi-synthetic opioid drug, which belongs to opioid receptor agonist. It can act on opioid δ receptor and μ receptor to achieve analgesic effect. It can also regulate the absorption and release of neurotransmitters, effectively reduce patient stress reactions, improve patient inflammation levels, and promote more stable hemodynamic fluctuations [20,21]. From the perspective of ventilation tools, compared with the laryngeal mask airway, the nasopharynx airway in the research group is a kind of non-endotracheal tube airway with soft material and placed outside the acoustic door. It has the advantages of shallow insertion depth and less stimulation. Compared with the laryngeal mask, it does not cause irritation to the tracheal mucosa or compression of the airbag. Besides, patients have good tolerance to nasopharynx airway, which helps maintain a stable internal environment and effectively alleviate stress reactions. In addition, some scholars believe that the use of nasopharyngeal airway-assisted ventilation is convenient for anesthesiologists to manage the patient’s breathing and circulation during surgery, thereby reducing the risk of fluctuations, maintaining relative stability of patient hemodynamic indicators, and alleviating stress reactions [22].

The research results also showed that there was no significant difference in retention time and postoperative recovery time between the two groups of patients. There was no significant difference in the number of intraoperative SpO_2_ < 92%, body movement response, and unplanned tracheal intubation between the two groups of patients, further indicating that both ventilation modes of general anesthesia can achieve good anesthesia effects. It is worth mentioning that there were two cases of tongue falling back in the research group, resulting in SpO_2_ < 92%, and SpO_2_ immediately increased to more than 98% after mandible support. In the control group, there was one case of displacement of the laryngeal mask. After adjusting the position and deepening anesthesia, ventilation was still unable to improve, leading to emergency tracheal intubation after SpO_2_ < 92%. The possible reasons may be that the laryngeal mask will undergo force deformation after inflation and there is no easily fixed physical structure. Due to factors such as the depth of anesthesia and changes in patient positioning, there is a risk of rotation and displacement, which can lead to inadequate ventilation and decreased pulse oximetry. The research group exhibited a significantly shorter airway establishment time compared to the control group. The possible reason is that the laryngeal mask has poor airtightness combined with individual anatomical differences, making it prone to air leakage. After placement, the laryngeal mask may be necessary to adjust the position until there is no air leakage. The above analysis also indicates that the placement of the nasopharyngeal airway is easier and the operation is simpler and more feasible. Patients in the research group reported significantly higher levels of satisfaction compared to those in the control group. This is attributed to the fact that the placement of laryngeal mask often leads to postoperative pain and discomfort in the throat area. In contrast, patients exhibited greater tolerance toward nasopharyngeal airway. Nevertheless, the incidence of bleeding cases during general anesthesia with laryngeal mask was notably higher than that with nasopharyngeal airway. The reason is speculated that the placement of the laryngeal mask requires more physiological bending structures, and there is a certain pressure when inflating and sealing the laryngeal cavity. Due to individual differences in patients and inflating pressure, it is easy to cause mucosal damage [23].

In summary, general anesthesia under nasopharyngeal airway or laryngeal mask ventilation modes can meet the requirements of sedation and analgesia for this surgery, with comparable anesthesia effects, which is beneficial for patient hemodynamic stability and reducing stress reactions. However, the operation of intravenous general anesthesia under nasopharyngeal airway-assisted ventilation is simpler, which can further reduce postoperative mucosal damage and other adverse reactions, improve patient satisfaction, and has certain promotion and application value in clinical practice.
